# Psychosocial impact and self-esteem in patients seeking dental aesthetic treatment: a cross-sectional study using PIDAQ and RSES

**DOI:** 10.3389/fpsyg.2026.1745236

**Published:** 2026-01-30

**Authors:** Simge Gumus Ayaz, Gokce Kececi, Ozgun Kokoz Citaker

**Affiliations:** 1 Department of Restorative Dentistry, Batman University, Batman, Türkiye; 2 Department of Prosthetic Dentistry, Kahramanmaras Sutcu Imam University, Kahramanmaras, Türkiye

**Keywords:** dental aesthetics, dental self-confidence, PIDAQ, psychosocial impact, Rosenberg self-esteem scale

## Abstract

**Background:**

The objective of our research is to find out how dental aesthetics affect people’s psychosocial status and self-esteem.

**Material and method:**

A total of 300 adult patients aged between 19 and 59 who presented to the Departments of Prosthetic Dentistry and Restorative Dentistry at Batman University Faculty of Dentistry for dental aesthetic treatment were included in this study. The Rosenberg Self-Esteem Scale (RSES) was used to measure self-esteem, while the Psychosocial Impact of Dental Aesthetics Questionnaire (PIDAQ) was employed to assess psychosocial status. Subgroup comparisons were performed using the Tukey *post-hoc* test following one-way analysis of variance (ANOVA) for multiple group analyses. Differences between gender groups were assessed using the independent samples *t*-test. The relationship between self-esteem and psychosocial variables was analyzed using Pearson’s correlation analysis.

**Results:**

No statistically significant difference was found between genders in terms of total PIDAQ scores; however, women had significantly lower RSES scores compared to men. A statistically significant difference was observed in PIDAQ total scores based on educational level (*p* < 0.05), with university graduates scoring significantly lower than high school graduates. Additionally, a statistically significant but weak positive correlation was found between total PIDAQ scores and RSES scores (*p* < 0.05; *r* = 0.323), indicating that as psychosocial impact scores increased, self-esteem levels also increased.

**Conclusion:**

The demand for dental aesthetic treatment is significantly associated with individuals’ psychological wellbeing and self-esteem. Therefore, when planning restorative and prosthetic treatments, it is essential to consider not only functional aspects but also the physical, social, and psychological dimensions of dental aesthetics.

## Highlights

- Dental aesthetic needs reflect not only functional but also psychological needs. Therefore, patients’ aesthetic expectations, along with their self-esteem and social interaction levels, should be considered in treatment planning.
- The lower self-esteem levels in women and the varying perception of aesthetics based on education level suggest that motivation for aesthetic treatment may vary depending on individual characteristics. This requires clinicians to provide personalized communication and counseling.
- The significant correlation between dental aesthetics and psychosocial status highlights that restorative and prosthetic treatments not only improve oral health but also have a positive impact on patients’ overall quality of life and social self-esteem.

## Introduction

The concepts of aesthetics encompassing smile and facial beauty are primarily based on the opinions of individuals rather than evidence-based scientific methods. However, due to the influence of beauty on diagnosis and treatment in dentistry, it is essential to define and measure this perception (Pinho et al., 2007). Dentofacial aesthetics is defined as the health of dental tissues and gingival tissue, their harmony, and the integrity of dental and gingival tissues during a smile (Reyneke and Ferretti, 2012).

A person’s smile is one of the first observable characteristics of the face, and the overall facial attractiveness is determined within a few seconds (Richards et al., 2015). Consequently, there has been a noticeable increase in the demand for treatments related to dentofacial aesthetics in recent times (Pachêco-Pereira et al., 2015; Yao et al., 2016). Research has shown that individuals with a regular dental appearance are perceived as more attractive, confident, and open to social interactions (Shaw et al., 1985). Aesthetic appearance, which is also thought to influence the perceptions of teachers and employers, has been associated with a higher likelihood of these individuals securing better job opportunities and advancing their careers (Pithon et al., 2014; Kenealy et al., 1991). Studies indicate that individuals with significant dentofacial deformities may face a form of social barrier (Chan et al., 2017; Seehra et al., 2011).

Orthodontics, restorative, and prosthetic treatment options are different disciplines that improve dentofacial aesthetics based on individual needs. The ultimate goal of treatment is to provide a lifelong positive psychosocial benefit, even though functional gains related to curing malocclusions and dental deformities may be limited in more severe cases (Wang et al., 2016; Radz, 2011). Recent studies indicate that more severe deformities draw greater attention to the oral region, making individuals more aware of dentofacial discrepancies rather than focusing on the nose and eyes (Dias and Gleiser, 2009; Liu et al., 2022). Therefore, understanding the psychosocial implications of aesthetic concerns has become increasingly important. Although several studies have explored dental aesthetics–related psychosocial outcomes, further research is needed in specific clinical populations seeking restorative and prosthetic treatment, who may differ in their aesthetic motivations and psychological responses. Additionally, the combined application of the PIDAQ and RSES scales provides a more comprehensive assessment of aesthetic self-perception and self-esteem, contributing novel insight to the existing literature.

This study aims to evaluate and determine the impact of the need for dental aesthetic treatment on psychosocial status and self-esteem in patients applying to restorative and prosthetic dentistry clinics. The null hypothesis of the study is that the desire for dental aesthetic treatment does not affect psychosocial status and self-esteem.

## Materials and methods

### Study design and participants

The research conducted was a cross-sectional study involving 300 patients who applied to the restorative dentistry and prosthetic dentistry departments of Batman University Faculty of Dentistry Hospital with a request for dental aesthetic treatment.

The data collection phase covered a four-month period from October 2024 to February 2025. The study was approved by the Non-Invasive Clinical Research Ethics Committee of Batman University (Date: October 3, 2024; Approval No: 2024/07).

To ensure privacy and minimize bias, participants completed the survey independently, without time constraints. No personal identifiers such as names or ID numbers were collected. All procedures were conducted in accordance with the principles of the Declaration of Helsinki, and participants were informed about the purpose and scope of the study prior to data collection.

### Sampling and sample size

The required sample size was determined through power analysis, assuming a 95% confidence level, 5% margin of error, and 80% statistical power, yielding a total of 300 participants. Participants were selected through convenience sampling method (Bleidorn et al., 2016).

This cross-sectional study was conducted in the restorative and prosthetic dentistry clinics of a university dental hospital. Patients aged 19–59 years who presented with a chief complaint related to anterior dental aesthetics and expressed a desire for dental aesthetic treatment were invited to participate. No dental aesthetic intervention was performed before questionnaire administration; all data were obtained at baseline.

### Inclusion criteria

- Individuals aged 19 to 59
- Those willing to participate
- Ability to understand and complete the questionnaires
- Presence of at least one anterior dental condition (e.g., discoloration, defective restoration, diastema, tooth fracture, or tooth loss) leading to a perceived need for aesthetic treatment.

### Exclusion criteria

- Individuals outside the 19–59 age range
- Those unwilling to participate
- Patients not requesting aesthetic anterior treatment
- History of cranial anomalies or syndromes
- Ongoing orthodontic treatment
- Previous orthognatic surgery
- Self-reported of severe psychiatric disorder.

Treatment needs were categorized according to the primary aesthetic indication rather than a standardized aesthetic severity index, which is acknowledged as a limitation in the Discussion.

The questionnaire consisted of three parts. The first section gathered demographic information (age, gender, marital status, education level) and the patient’s reason for seeking treatment. It also recorded complaints related to the anterior region and smile line classification. The second section included the 10-item Rosenberg Self-Esteem Scale (RSES), and the third section utilized the Psychosocial Impact of Dental Aesthetics Questionnaire (PIDAQ) to assess patients’ psychosocial outcomes related to dental aesthetics.

### Survey design

Data collection used standardized questionnaires measuring the Rosenberg Self-Esteem Scale (RSES) and the Psychosocial Impact of Dental Aesthetics (PIDAQ).

### Rosenberg self-esteem scale (RSES)

The Rosenberg Self-Esteem Scale (RSES), developed by Rosenberg (1965), is widely used to assess self-esteem levels. The Turkish validity and reliability study of the scale was conducted by Çuhadaroğlu in 1986 (Berberoğlu and Hocaoğlu, 2023). The original scale consists of 63 items grouped under 12 subcategories; however, only the first 10 items specifically related to self-esteem were used in this study.

The RSES uses a 4-point Likert-type response format with the options: A (Strongly Agree), B (Agree), C (Disagree), and D (Strongly Disagree), which are scored as 1, 2, 3, and 4, respectively. Scores were evaluated using the Guttman scaling method. A total score of 0–1 was interpreted as high self-esteem, 2–4 as moderate self-esteem, and 5–6 as low self-esteem. In this system, higher scores reflect lower levels of self-esteem, while lower scores indicate higher self-esteem (Davis et al., 2009; Çoban Büyükbayraktar and Doruk, 2021).

### Psychosocial impact of dental aesthetics questionnaire (PIDAQ)

The Psychosocial Impact of Dental Aesthetics Questionnaire (PIDAQ) was utilized to evaluate the participants’ psychosocial status (Klages et al., 2006). This validated psychometric instrument consists of 23 items divided into four subscales: dental self-confidence (6 items), social impact (8 items), psychological impact (6 items), and aesthetic concern (3 items) (Montiel-Company et al., 2013). Each item is rated on a 5-point Likert scale ranging from 1 (Strongly Disagree) to 5 (Strongly Agree). Items in the dental self-confidence subscale are reverse-scored.

Since its development, PIDAQ has undergone reliability and validity testing and has been adapted into various languages (Lin et al., 2013; Sardenberg et al., 2011). Aglarci et al. (2016) conducted the Turkish validity and reliability study of the scale. Prior to the current study, permission to use the PIDAQ was obtained via email from Prof. Dr. Ulrich Klages.

### Survey distribution

The final version of the survey was distributed to patients by specialist dentists in restorative and prosthetic dentistry clinics. In addition to the survey, participants were given a cover letter informing them about the aims of the study and participant confidentiality, and their consent was obtained. Participation in the research was voluntary, and no financial compensation was paid. Participants could withdraw at any time.

### Statistical analysis

The data were analyzed using the licensed IBM SPSS Statistics 21 software package. To assess whether the variables followed a normal distribution, the Shapiro–Wilk and/or Kolmogorov-Smirnov tests were performed depending on the sample size. A significance level of 0.05 was used for all statistical evaluations. If the *p*-value was less than 0.05, the data were considered not normally distributed; otherwise, they were assumed to follow a normal distribution.

Since the variables did not meet the assumption of normality, non-parametric tests were applied. The Mann–Whitney U test was used for comparisons between two groups, and the Kruskal-Wallis H test was used for comparisons among more than two groups. When the Kruskal-Wallis H test indicated significant differences, a *post-hoc* multiple comparison test was performed to determine which specific groups differed.

Spearman’s rank-order correlation coefficient was used to assess the relationships between variables that were not normally distributed. Throughout the analysis, a *p*-value of less than 0.05 was considered statistically significant.

## Results

Among the study participants, 40.67% were female and 59.33% were male. Regarding age distribution, 39% were between 19 and 29 years old, 36% were between 30 and 39, 18.67% were between 40 and 49, and 6.33% were between 50 and 59.

In terms of educational attainment, 13.67% had completed primary school, 30% had completed high school, 49.67% were university graduates, and 6.67% held a master’s or doctoral degree.

Marital status analysis revealed that 60.33% of the participants were married, while 39.67% were single. Regarding smile line classification, 58.67% had a normal smile line, and 23.33% presented with a high smile line.

A statistically significant difference was found between genders in terms of the Rosenberg Self-Esteem Scale (RSES) scores (*p* < 0.05), with female participants scoring significantly lower than males. However, no statistically significant difference was observed between genders in the total PIDAQ scores (*p* > 0.05) (Table 1).

**Table 1:** Findings from an analysis of the differences between genders on the PIDAQ and RSES.

RSES and PIDAQ scales		Gender						Mann Whitney U test		
		*n*	Mean	Median	Min	Max	Std	Mean rank	*z*	*p*
RSES	Woman	122	1.84	2	1	3	0.5	139.91	−2.272	**0.023**
	Men	178	1.97	2	1	3	0.5	157.76		
	Total	300	1.92	2	1	3	0.51			
Dental confidence	Woman	122	15.23	14.5	6	30	5.45	140.98	−1.578	0.115
	Men	178	16.19	16	6	30	5.01	157.03		
	Total	300	15.8	16	6	30	5.2			
Social impact	Woman	122	16.33	15.5	8	32	5.62	142.58	−1.311	0.19
	Men	178	17.26	16	8	34	6.08	155.93		
	Total	300	16.88	16	8	34	5.9			
Psychological impact	Woman	122	14.63	14	6	27	4.68	143.66	−1.133	0.257
	Men	178	15.19	15	6	29	4.58	155.19		
	Total	300	14.96	15	6	29	4.62			
Aesthetic anxiety	Woman	122	6.96	6	3	13	2.53	144.21	−1.049	0.294
	Men	178	7.33	7	3	15	2.71	154.81		
	Total	300	7.18	7	3	15	2.64			
PIDAQ total	Woman	122	53.15	50	23	91	14.75	139.31	−1.851	0.064
	Men	178	55.97	55	30	108	14.26	158.17		
	Total	300	54.82	53	23	108	14.5			

Bold values indicate *p* < 0.05.

A statistically significant difference was found in Social Impact scores according to educational status (*p* < 0.05), with university graduates exhibiting significantly lower scores compared to primary and high school graduates.

Similarly, Psychological Impact scores differed significantly by educational level (*p* < 0.05). University graduates had significantly lower scores than primary school graduates.

In terms of the total PIDAQ score, a significant difference was also observed between education groups (*p* < 0.05). Specifically, university graduates had significantly lower total PIDAQ scores than high school graduates (Table 2).

**Table 2:** Relationship between educational status and RSES and PIDAQ scale.

RSES and PIDAQ scales		Education status						Kruskal Wallis H test		
		*N*	Mean	Median	Min	Max	Std	Mean rank	H	*p*
RSES	Primary education	41	1.9	2	1	3	0.58	148.16	2.757	0.431
	High school graduates	90	1.99	2	1	3	0.57	159.54		
	University graduates	149	1.87	2	1	3	0.44	145.09		
	Master’s/doctorate graduates	20	1.95	2	1	3	0.51	154.93		
	Total	300	1.92	2	1	3	0.51			
Dental confidence	Primary education	41	14.63	15	7	24	4.65	130.68	5.402	0.145
	High school graduates	90	16.5	17	6	25	4.76	165.52		
	University graduates	149	15.63	15	6	30	5.46	145.94		
	Master’s/doctorate graduates	20	16.3	16.5	6	30	6.04	157.5		
	Total	300	15.8	16	6	30	5.2			
Social impact	Primary education	41	18.44	17	12	32	4.77	179.11	15.796	**0.001**
	High school graduates	90	18.11	17	8	33	6.05	169.48		
	University graduates	149	15.69	15	8	32	5.76	131.83		
	Master’s/doctorate graduates	20	17	16	8	34	6.88	145.57		
	Total	300	16.88	16	8	34	5.9	3-2 3-1		
Psychological impact	Primary education	41	16.37	16	6	27	3.94	183.06	9.172	**0.027**
	High school graduates	90	14.94	15	6	26	4.44	153.86		
	University graduates	149	14.45	14	6	28	4.73	138.24		
	Master’s/doctorate graduates	20	16	14.5	10	29	5.4	159.98		
	Total	300	14.96	15	6	29	4.62	3-1		
Aesthetic anxiety	Primary education	41	6.95	7	3	13	2.62	142.44	1.589	0.662
	High school graduates	90	7.22	6	3	15	2.68	150.99		
	University graduates	149	7.13	7	3	15	2.61	149.58		
	Master’s/doctorate graduates	20	7.8	7.5	3	15	2.89	171.63		
	Total	300	7.18	7	3	15	2.64			
PIDAQ total	Primary education	41	56.39	55	31	91	13.02	162.52	8.077	**0.044**
	High school graduates	90	56.78	57	23	95	13.81	167.23		
	University graduates	149	52.91	50	30	98	14.54	136.49		
	Master’s/doctorate graduates	20	57.1	54	34	108	18.88	154.93		
	Total	300	54.82	53	23	108	14.5	3-2		

Bold values indicate *p* < 0.05.

**Figure 1:**
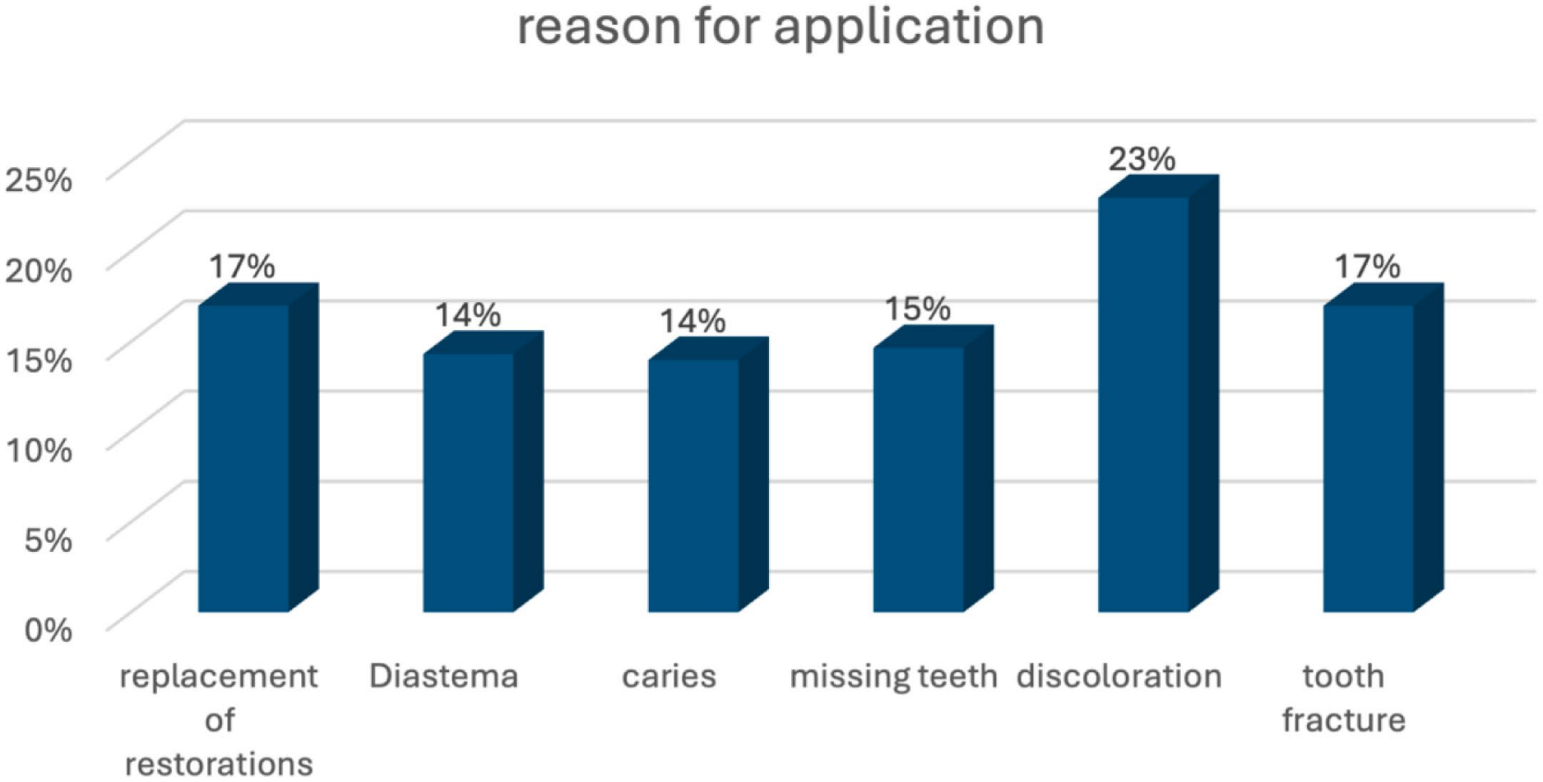
Distribution of participants according to reasons for application.

Statistically significant differences were observed in Rosenberg Self-Esteem Scale (RSES) scores based on the reasons for application (*p* < 0.05). Individuals presenting with missing teeth had significantly lower self-esteem scores compared to other groups (see Figure 1).

Dental Self-Confidence scores also varied significantly by reason for application (*p* < 0.05). Those applying due to missing teeth had the lowest scores among all groups. Additionally, applicants with caries reported significantly lower scores than those seeking treatment for restoration replacement or discoloration, while individuals with tooth fractures scored lower than those with restoration replacement needs.

In terms of Social Impact scores, a significant difference was found (*p* < 0.05). Applicants with caries or missing teeth reported significantly lower scores than those presenting for restoration replacement.

Psychological Impact scores also showed significant differences (*p* < 0.05), with those seeking treatment for caries reporting lower scores than those applying for restoration replacement.

Aesthetic Anxiety scores differed significantly between groups (*p* < 0.05). Individuals presenting with diastema or caries had lower scores compared to those requiring restoration replacement.

Finally, total PIDAQ scores demonstrated significant variation by application reason (*p* < 0.05). Applicants with missing teeth scored significantly lower than those with discoloration, restoration replacement, or tooth fracture. Those presenting with caries also had lower total scores than individuals with discoloration or restoration needs, and participants with diastema or tooth fracture scored lower than those with restoration replacement (Table 3).

**Table 3:** Analysis of differences between application reasons in terms of RSES and PIDAQ.

RSES and PIDAQ scales		Reason for application						Kruskal Wallis H Test		
		*n*	Mean	Median	Min	Max	Std	Mean rank	H	*P*
RSES	Replacement of restorations	51	2.2	2	1	3	0.45	187.07	146.448	**0.001**
	Diastema	43	1.93	2	1	3	0.4	152.9		
	Caries	42	1.93	2	1	3	0.34	152.95		
	Missing teeth	44	1.11	1	1	3	0.39	41.73		
	Discoloration	69	2.04	2	1	3	0.32	168.03		
	Tooth fracture	51	2.14	2	2	3	0.35	180.02		
	Total	300	1.92	2	1	3	0.51	4-2 4-3 4-5 4-6 4-1		
Dental confidence	Replacement of restorations	51	20.08	20	12	30	4.72	218.47	120.237	**0.001**
	Diastema	43	15.91	15	7	30	4.77	152.66		
	Caries	42	13.88	13	6	24	3.47	116.12		
	Missing teeth	44	9.11	8.5	6	19	2.7	39.11		
	Discoloration	69	17.38	17	9	25	4.14	179.36		
	Tooth fracture	51	16.65	16	10	29	3.97	166.07		
	Total	300	15.8	16	6	30	5.2	4-2 4-3 4-5 4-6 4-1 3-5 3-1 2-1 6-1		
Social impact	Replacement of restorations	51	19.55	18	8	34	6.56	185.64	16.36	**0.006**
	Diastema	43	16.74	16	8	32	5.86	149.66		
	Caries	42	14.69	14.5	8	26	4.43	120.8		
	Missing teeth	44	15.95	14.5	8	33	6.03	131.14		
	Discoloration	69	17.25	17	8	32	5.71	158.98		
	Tooth fracture	51	16.43	16	8	29	5.69	145.76		
	Total	300	16.88	16	8	34	5.9	3-1 4-1		
Psychological impact	Replacement of restorations	51	16.76	16	7	29	4.98	180.25	14.475	**0.013**
	Diastema	43	14.4	14	6	24	3.97	141.7		
	Caries	42	13.5	13	7	22	3.93	121.05		
	Missing teeth	44	13.82	13.5	6	23	4.24	131.52		
	Discoloration	69	15.52	15	6	27	4.83	159.8		
	Tooth fracture	51	15.08	15	6	26	4.76	156.22		
	Total	300	14.96	15	6	29	4.62	3-1		
Aesthetic anxiety	Replacement of restorations	51	8.24	8	3	15	2.92	181.49	15.223	**0.009**
	Diastema	43	6.35	6	3	12	2.35	124.98		
	Caries	42	6.5	6	3	13	2.57	128.06		
	Missing teeth	44	7.52	7.5	3	13	2.6	164.6		
	Discoloration	69	7.32	7	3	15	2.75	154.91		
	Tooth fracture	51	6.9	6	3	12	2.19	141.38		
	Total	300	7.18	7	3	15	2.64	2-1 3-1		
PIDAQ total	Replacement of restorations	51	64.63	63	31	108	15.63	206.27	47.501	**0.001**
	Diastema	43	53.4	53	31	98	13.27	143.16		
	Caries	42	48.57	46	31	79	11.23	111.13		
	Missing teeth	44	46.41	45.5	23	81	11.93	99.99		
	Discoloration	69	57.46	55	33	91	14.25	166.53		
	Tooth fracture	51	55.06	55	33	89	12.61	155.23		
	Total	300	54.82	53	23	108	14.5	4-6 4-5 4-1 3-5 3-1 2-1 6-1		

Bold values indicate *p* < 0.05.

Statistically significant differences were observed between smile line groups in terms of Rosenberg Self-Esteem Scale scores (*p* < 0.05). Individuals with a high smile line had significantly lower self-esteem scores compared to those with normal or low smile lines.

Regarding dental self-confidence, the differences among smile line groups were also statistically significant (*p* < 0.05). Participants with a high smile line had significantly lower dental self-confidence scores than those with normal or low smile lines, while individuals with a low smile line also scored lower than those with a normal smile line.

For the social impact domain, a significant difference was found between groups (*p* < 0.05), with the high smile line group exhibiting significantly lower scores compared to the normal and low smile line groups.

Psychological impact scores also showed a statistically significant difference between smile line groups (*p* < 0.05). Specifically, individuals with a high smile line reported significantly lower psychological impact scores than those with a normal smile line.

Lastly, the total PIDAQ score varied significantly according to smile line classification (*p* < 0.05), with the high smile line group reporting significantly lower scores compared to both the normal and low smile line groups (see Table 4).

A statistically significant positive correlation was found between dental self-confidence and RSES scores (*p* < 0.05), with a moderate strength of association (*r* = 0.500). As dental self-confidence increases, self-esteem also tends to increase.

A weak but statistically significant positive correlation was observed between Social Impact and RSES scores (*p* < 0.05; *r* = 0.218), indicating that higher social impact scores are associated with higher self-esteem.

Similarly, a weak positive correlation was found between Psychological Impact and RSES scores (*p* < 0.05; *r* = 0.146), suggesting that increases in psychological impact are related to higher self-esteem levels.

**Table 4:** Analysis of the difference between the smile line on the RSES and PIDAQ scales.

RSES and PIDAQ scales		Smile line						Kruskal Wallis H Test		
		*n*	Mean	Median	Min	Max	Std	Mean rank	H	*P*
RSES	Normal	176	2.11	2	1	3	0.4	176.74	112.281	**0.001**
	High	70	1.37	1	1	2	0.49	77.2		
	Low	54	1.98	2	1	3	0.31	160.01		
	Total	300	1.92	2	1	3	0.51	2-3 2-1		
Dental confidence	Normal	176	18.09	18	8	30	4.69	188.82	99.302	**0.001**
	High	70	10.9	10	6	22	3.87	68.89		
	Low	54	14.69	14.5	9	25	3.19	131.4		
	Total	300	15.8	16	6	30	5.2	2-3 2-1 3-1		
Social impact	Normal	176	17.74	17	8	34	6.05	163.67	17.814	**0.001**
	High	70	14.54	14	8	30	5	112.54		
	Low	54	17.09	17	8	33	5.77	156.79		
	Total	300	16.88	16	8	34	5.9	2-3 2-1		
Psychological impact	Normal	176	15.85	15	6	29	4.88	165.43	15.55	**0.001**
	High	70	13.1	13	6	21	3.83	117.75		
	Low	54	14.5	14	6	24	3.89	144.28		
	Total	300	14.96	15	6	29	4.62	2–1		
Aesthetic anxiety	Normal	176	7.3	7	3	15	2.64	154.01	1.822	0.402
	High	70	7.26	7	3	15	2.76	152.66		
	Low	54	6.7	6	3	15	2.5	136.25		
	Total	300	7.18	7	3	15	2.64			
PIDAQ total	Normal	176	58.98	56.5	31	108	15	174.18	41.373	**0.001**
	High	70	45.8	44	23	69	10.28	95.68		
	Low	54	52.98	53	31	81	11.73	144.38		
	Total	300	54.82	53	23	108	14.5	2-3 2-1		

Bold values indicate *p* < 0.05.

Finally, a statistically significant weak positive correlation was identified between total PIDAQ scores and RSES scores (*p* < 0.05; *r* = 0.323). This indicates that as individuals’ overall PIDAQ scores increase, their self-esteem levels also increase (Table 5).

**Table 5:** Analysis results of the relationship between the RSES and the PIDAQ scale.

RSES and PIDAQ scales		RSES
Dental confidence	*r*	0.500**
	*p*	**0.001**
	*n*	300
Social impact	*r*	0.218**
	*p*	**0.001**
	*n*	300
Psychological impact	*r*	0.146*
	*p*	**0.011**
	*n*	300
Aesthetic anxiety	*r*	−0.028
	*p*	0.632
	*n*	300
PIDAQ total	*r*	0.323**
	*p*	**0.001**
	*n*	300

*,**This relationship is in the same direction and of weak strength. Bold values indicate *p* < 0.05.

## Discussion

The concept of aesthetics, defined as the care that an individual shows for his/her appearance, is an important factor that has a direct effect on psychology. Dental and smile aesthetics are among the components that contribute greatly to appearance. Dental and smile aesthetics, which are an important part of facial aesthetics, affect people’s relationships with other people in society and themselves. Having an aesthetic smile provides an increase in self-confidence and comfort in social relationships (Manera et al., 2013). In our study, it was concluded that the desire for dental aesthetic treatment has an effect on psychosocial status and self-esteem. Consequently, the study’s null hypothesis was disproved.

In dentistry, different scales have been utilized to measure the effect of dental problems on the quality of life of individuals. Among the scale examples in the literature, Turkish validity/reliability studies have been conducted for Oral Impact on Daily Performances (OIDP), Oral Health Impact Profile (OHIP), and PIDAQ scales. A customized scale called the PIDAQ was created to assess the psychological impacts of dental aesthetics on young adults. Validity/reliability studies of the PIDAQ scale, which is accepted to have high validity among the scales used in dentistry, have been conducted in various languages (Klages et al., 2015; Bourzgui et al., 2015; Santos et al., 2016). The validity and reliability study of the Turkish adaptation of this scale was published by Aglarci et al. (2016). In all studies conducted on this subject, PIDAQ was evaluated within the scope of orthognathic surgery and orthodontic treatment. The originality value of this study is that, unlike previous studies, adult patients who applied to prosthodontic and restorative dental treatment clinics with the demand for aesthetic treatment were also included. To date, PIDAQ has rarely been applied in populations seeking restorative or prosthodontic aesthetic treatment. Thus, this study represents one of the first attempts to apply the PIDAQ scale within this specific clinical context, providing novel data from a population that has not been examined in previous research.

The simultaneous use of both PIDAQ and RSES offers a more comprehensive evaluation of patients’ psychosocial profiles, integrating dental aesthetic perception with global self-esteem. This combined approach provides a level of depth not present in earlier studies, thereby enhancing the originality and contribution of this research. In addition to being specific to self-esteem and unidimensional, it has important advantages such as ease of administration, scoring, and interpretation, as well as high internal consistency, validity, and reliability. Furthermore, this scale is accepted as the gold standard in developing other scales (Robinson et al., 2013). Due to these reasons, the RSES was preferred in our study, and self-esteem was specifically evaluated in terms of the need for restorative and prosthetic treatment, which has not been addressed in the literature before.

Chen et al. included individuals aged 35–56 years in their study. In this study, patients between the ages of 19–59 who needed aesthetic treatment in their anterior teeth were included in the study with the idea that the answers given in different age groups would also differ (Chen et al., 2012).

In the current literature, no relationship was found between genders regarding self-esteem (Çoban Büyükbayraktar and Doruk, 2021; Stojilković et al., 2024). In our study, the self-esteem of women was found to be lower, and these results were in agreement with a study conducted. According to researchers this difference may lead to stronger or weaker reactions of people of different genders to negatively expressed options and thus to differences in mean self-esteem scores (Bleidorn et al., 2016). No psychosocial difference was found between genders, which is in parallel with an another study (DiStefano and Motl, 2009).

In our study, it was concluded that the level of education had an effect on psychosocial status. Although this result coincides with current studies, it is in conflict with Poyraz’s study (Chen et al., 2012; Poyraz, 2018; Üstün et al., 2013; Wahab et al., 2021; Rai et al., 2021). In addition, a significant correlation was found between education level and self-esteem in our study. These results draw attention to the awareness of the links between dental appearance and social status in patients with higher education levels.

In this study, patient selection was made by considering different aesthetic indications. The PIDAQ scores of patients presenting with tooth deficiency were significantly lower than those presenting with discoloration, replacement of restorations, and tooth fracture, and the PIDAQ total score of patients presenting with diastema and tooth fracture was significantly lower than that of patients presenting with restoration renewal. These results were similar to those of Poyraz and Dahong (Poyraz, 2018; Dahong et al., 2013; Ghorbani et al., 2025)_._

The results of our study showed that the level of the smile line can affect an individual in terms of self-esteem and psychosocial aspects. According to the smile line, there was a significant difference between the groups’ PIDAQ scores on the subscales measuring aesthetic anxiety, dental self-confidence, social influence, and self-esteem. Self-esteem and psychosocial scores of patients with high smile lines were found to be significantly lower, and these results partially coincide with current studies (Poyraz, 2018).

We also assessed the correlation between the PIDAQ Scale, which we employed in our study, and the RSES. In the present study, a significant and positive correlation was observed between the psychosocial effect of dental aesthetics and self-esteem. Similar findings were seen by Venete and Stojilković, suggesting a positive correlation between self-esteem and the PIDAQ dental self-confidence subdomain (Stojilković et al., 2024; Venete et al., 2017). Accordingly, people who are more impacted by oral aesthetics typically have lower self-esteem.

### Limitations

This study has several limitations that should be considered when interpreting the findings. First, its cross-sectional design precludes any causal inference between the perceived need for dental aesthetic treatment and psychosocial outcomes. Second, we did not use a standardized index to quantify the severity of dental aesthetic impairment; instead, patients were categorized according to their main aesthetic indication (discoloration, restoration replacement, diastema, fracture, or tooth loss). This approach may have introduced residual confounding. Third, systemic health conditions, subclinical psychological disorders, and explicit satisfaction with current dental appearance were not assessed with specific standardized measures and could have influenced both PIDAQ and RSES scores. Therefore, the associations reported in this study should be interpreted with caution and considered hypothesis-generating, particularly for patients seeking restorative and prosthetic aesthetic treatment.

## Conclusion

The findings of this study reveal a significant linear relationship between the need for dental aesthetic treatment and individuals’ self-esteem and psychosocial wellbeing. These results emphasize the importance of considering not only functional aspects such as mastication, phonation, and overall oral function, but also the physical, social, and psychological dimensions of dental aesthetics when planning restorative and prosthetic treatments.

## Data Availability

The original contributions presented in the study are included in the article/supplementary material, further inquiries can be directed to the corresponding author.
